# Is There a Future for Nuclear Power? Wind and Emission Reduction Targets in Fossil-Fuel Alberta

**DOI:** 10.1371/journal.pone.0165822

**Published:** 2016-11-30

**Authors:** G. Cornelis van Kooten, Jun Duan, Rachel Lynch

**Affiliations:** Department of Economics. University of Victoria, Victoria, Canada; University of Liverpool, UNITED KINGDOM

## Abstract

This paper explores the viability of relying on wind power to replace upwards of 60% of electricity generation in Alberta that would be lost if coal-fired generation is phased out. Using hourly wind data from 17 locations across Alberta, we are able to simulate the potential wind power output available to the Alberta grid when modern, 3.5 MW-capacity wind turbines are spread across the province. Using wind regimes for the years 2006 through 2015, we find that available wind power is less than 60% of installed capacity 98% of the time, and below 30% of capacity 74% of the time. There is only a small amount of correlation between wind speeds at different locations, but yet it remains necessary to rely on fossil fuel generation. Then, based on the results from a grid allocation model, we find that CO_2_ emissions can be reduced by about 30%, but only through a combination of investment in wind energy and reliance on purchases of hydropower from British Columbia. Only if nuclear energy is permitted into the generation mix would Alberta be able to meet its CO_2_-emissions reduction target in the electricity sector. With nuclear power, emissions can be reduced by upwards of 85%.

## Introduction

The Government of Alberta promised to make the province a world leader in renewable energy. To achieve this, all coal-fired electricity generation facilities are to be phased out by 2030, with two-thirds of the lost electricity production to be replaced by renewables, primarily wind and solar power, with natural gas to be used for generating baseload power (and as backup to intermittent energy sources). To encourage a speedy transition, the government will implement an economy-wide carbon tax of $20 per tonne CO_2_ (tCO_2_) beginning in 2017 and increase it to $30/tCO_2_ in 2018; commit to produce 30% of electricity from renewables by 2030; provide subsidies to encourage renewable energy; and cap emissions from oil sands developments at 100 megatons of CO_2_ [1].

The purpose of the current study is to investigate whether it would be possible for Alberta to reduce its CO_2_ emissions by 30% or more, replacing two-thirds of the lost coal-fired power with wind generated electricity. To do so, we employ a grid optimization model that enables investment and disinvestment in generating facilities, and ensure that there might be sufficient wind at any given time by employing uncorrelated wind regimes from various sites throughout the province. The analysis is at macro scale since the objective is to guide policy related to climate change rather than prescribe actual investment decisions regarding generating assets or transmission requirements.

We begin in the next section by examining the characteristics of the Alberta electricity system, followed by a discussion of the costs of producing electricity. Then we describe our model and the origins of the wind power data that we employ. This is followed by our results and concluding discussion. Our results indicate that, for Alberta to reduce CO_2_ emissions from the production of electricity by 30% or more will likely require something more than investments in wind energy, with nuclear energy looking most promising despite its high costs.

## Background

The costs of developing and operating renewable (wind- or solar-powered) generating assets can be substantial and may only be viable for firms when governments provide subsidies or other inducements. An indirect but important cost of these renewables is associated with the high variability of wind patterns and inconsistent availability of solar energy (due to lack of sunlight). Intermittency in wind (and solar) power output is unavoidable, often resulting in large costs of ramping existing generating assets or investing in new assets to compensate for this intermittency [2]. Nonetheless, there are also significant benefits to society of transitioning away from fossil fuels, primarily from reduced greenhouse gas (GHG) emissions as measured in terms of CO_2_. The benefits to society relate mainly to the substitution of clean energy for fossil fuels, especially coal-fired power.

We focus only on wind power because potential solar photovoltaic (PV) electricity output is more difficult to model and beyond the scope of the current study. However, the inherent intermittency we model using wind is likely little affected by adding solar power. For example, [3] found that adding a predictable tidal power output profile to a wind profile had no impact on the management of a power grid on Haida Gwaii off the Northwest coast of British Columbia.

To measure the degree to which intermittent energy can substitute for coal-fired power will be determined in this study by first collecting hourly wind data from various locations across the Alberta. We then assume establishment of sufficient wind generating capacity to meet half of the province’s peak load, with wind farms spread across the province to reduce the potential intermittency in supply as wind speeds vary across the landscape. We simulate the wind power that could have been generated every hour for the period 2006 through 2015 using wind-turbine power curves and the data on wind speeds. Hourly wind power output is then subtracted from demand to obtain the load that must be met by the various fossil-fuel and other generating assets comprising the Alberta electricity system.

Our objective is to examine the case where the Alberta government seeks to eliminate coal, using both a carbon tax and regulation. To determine how generation is allocated across assets in each hour, we use an existing grid model for Alberta that optimizes load across assets [4]. The grid allocation model is annual with an hourly time step, and assumes rational expectations on the part of the grid operator/asset owner. Any excess power remaining when intermittent wind power is subtracted from load is assumed to be exported to British Columbia, which has the ability to store power behind hydroelectric dams, or to Saskatchewan or the United States via transmission interties. Excess power is assumed to be sold at a price of zero–Alberta receives no remuneration nor does it have to pay (negative price) to dump the excess power. Costs are determined in the analysis using the levelized cost of electricity (LCOE) when power is generated from various facilities as determined by the grid allocation model, although an investor is assumed to incur an annualized cost of construction. The optimal allocation of output across assets is also used to determine CO_2_ emissions.

Our analysis seeks to determine if the benefits to society from changing to wind and solar energy will outweigh the costs, and whether imposing strong restrictions on fossil fuels is economically feasible. We can calculate the costs and benefits of reducing CO_2_ emissions from Alberta’s electricity sector; in doing so, we rely on assumed values of the social cost of carbon or price of carbon (see [5]; [6]). Depending on the results, it may be necessary to examine the optimal subsidies required by governments to facilitate the construction of renewable generating facilities as well as the compensation for firms that had earlier been encouraged to invest in new coal-fired generating capacity.

### Alberta Electricity Grid

**Fig 1:**
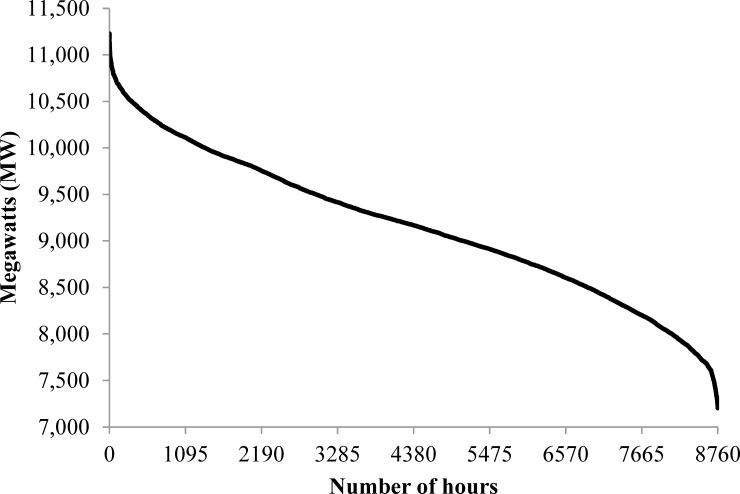
Alberta Load Duration Curve, 2015.

**Fig 2:**
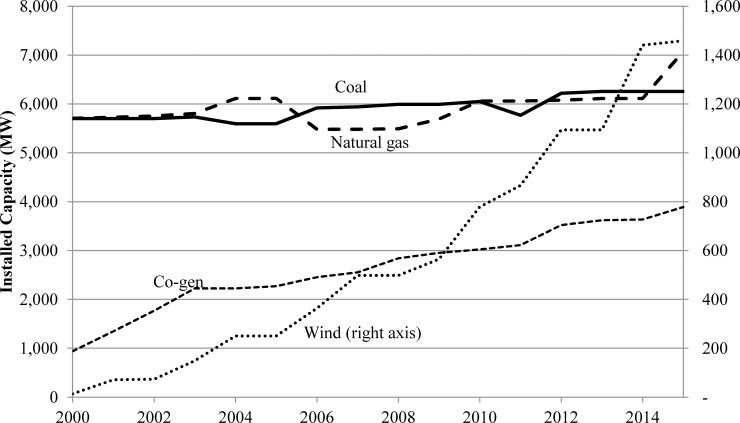
Installed Generating Capacity by Type, Alberta, 2000–2015.

The Alberta electricity grid is characterized by industrial consumers and three main types of generation–coal, natural gas and co-generation. The 2015 load duration curve shown in Fig 1 is indicative of the province’s industrial base; the peak load of 11,229 MW is only 56% greater than the baseload of 7,203 MW, and baseload demand (63.10 TWh) accounts for 78.6% of total generation of 80.26 TWh. In contrast, for example, the peak loads of British Columbia and Ontario are more than double those of baseload. Alberta’s generation mix is dominated by fossil fuels despite recent efforts to increase use of biomass and wind (there is no solar in the mix), and recovery of waste heat. As indicated in Fig 2, installed wind capacity has increased by 1,445 MW since 2000, while co-generation capacity increased by 2,951 MW (most of which relies on natural gas and not biomass), natural gas plant capacity by 1,372 MW, and coal-fired capacity by 556 MW, although overall investment in coal capacity has been greater since over 500 MW was decommissioned during the same period.

### Costs of Producing Electricity

A recent study by [7] examined the costs of generating electricity in the U.S. by three types of assets: baseload assets capable of dispatching electricity at any time and for very long periods (coal, combined-cycle natural gas, nuclear and hydro), dispatchable peak resources (gas turbines), and intermittent resources (wind). The authors compared [8] estimates of the LCOE based on information from existing plants, estimates of what it would cost to produce electricity from new plants with the latest technology, and estimates for new construction but revised to take into account observed capacity factors (CFs) rather than assumed CFs. A generating asset’s capacity factor is given by the ratio of the annual electricity generated by the asset divided by the asset’s capacity multiplied by 8760 hours (8784 hours in a leap year). Stacy and Taylor’s LCOE calculations are provided in Table 1, along with more recent estimates from [9].

**Table 1:** Estimates of the Levelized Costs of Electricity (LCOE) for Existing Plants, New Construction with Optimistic Capacity Factors and New Construction based on Observed Capacity Factors, and Latest Estimates, Three Generating Asset Types ($US 2012/MWh).

Generator Type	Existing^a^	Optimistic^a^	Observed^a^	Latest^b^
**Dispatchable full-time capable resources (baseload)**				
Conventional coal	38.4	80.0	97.7	93.7
Conventional combined cycle gas (CC gas)	48.9	66.3	73.4	74.1
Nuclear	29.6	96.1	92.7	93.8
Hydro (seasonal)	34.2	84.5	116.8	82.2
**Dispatchable peaking resources**				
Conventional combustion turbine (CT gas)	142.8	128.4	362.1	139.4
**Non-dispatchable intermittent resource as used in practice**				
Wind	Not available	96.2	112.8	72.5

^a^ Source:[7]. The ‘Existing’ column is based on their own calculations. Data in the ‘Optimistic’ and ‘Observed’ columns are based on [8].

^b^ Source: [9]. Values for plants entering service in 2020; $2013 values deflated to $2012 using inflation rate of 1.5%. Capacity factors for wind (36%) and solar (25%) are the best observed in the U.S., so LCOEs for intermittent resources are likely higher than reported here.

The results in Table 1 indicate that current costs of producing electricity (Existing column) are much lower than those of new construction. This is primarily because the construction costs of many assets have been paid off. Decision makers need to consider this when they implement policies that result in the premature closure of existing generators, because doing so might lead to higher than expected overall electricity costs. Next, estimates of the LCOEs for new construction indicate that, despite recent advances in technology, wind remains at a cost disadvantage relative to fossil fuels, and more so if costs of additional transmission are taken into account. Finally, if the observed as opposed to estimated CF is used to calculate levelized costs, the LCOE for new construction will turn out to be higher than expected by the EIA (2010, 2015), thereby reinforcing preference for keeping current assets longer.

There are two caveats to consider. First, the use of LCOE to select renewable energy projects (or otherwise make investment choices) can be misleading because the value of power changes over time and space, as does the production of power from various assets, especially intermittent ones. Second, LCOE estimates exclude externality costs, except perhaps in the case of nuclear power, where recent cost overruns to address evolving environmental regulations have resulted in construction delays and higher costs [10]. This issue is best addressed by employing an annualized cost (or penalty) for investing in new generating capacity, which could be over and above the carbon tax used to incentivize both investment and generation.

In the current application, the annualized costs are determined by assuming that the lifetimes of all generating assets are the same (30 years). This disadvantages investments in nuclear and coal compared to other assets, because the construction cost is spread over fewer years. In the case of coal, however, the carbon tax ensures that no further capacity is added. Finally, a (quite) small penalty is imposed to incentivize removal of assets that fail to produce power during the year.

Overnight construction costs are difficult to determine. In the current analysis, we use data from surveys conducted at various times by the International Energy Agency (IEA) and U.S. Energy Information Administration (EIA). It should be noted, however, that our results are robust regarding capital costs (e.g., see http://www.eia.gov/oiaf/beck_plantcosts/index.html [accessed September 8, 2016]). We assume overnight costs of wind are $2,700 per kW, while those of coal, conventional combustion turbine (CT) gas (which we assume to be the same as for co-gen), combined-cycle (CC) gas, and nuclear power plants are $2,600, $1,900, $1,600 and $6,000 per kW of installed capacity, respectively. Capital costs are annualized using a 5% discount rate and the estimated length of time taken to build the facility, which are taken to be 7 years for nuclear power plants, 4 years for coal assets, 2 years of CT gas, and 3 years for co-gen/CC gas facilities.

Wind power is less expensive on a LCOE basis than CT gas and seasonal hydro, but more expensive than electricity generated from CC gas, nuclear and coal. Overall, available data indicate that traditional fossil fuel technologies are clearly preferred to wind power on a cost basis, unless externality costs are taken into account.

## Alberta Model

The Alberta grid allocation model is described in various places; here we provide a brief description as found in [4]. The Alberta Electric System Operator (AESO) is considered to be the decision maker, so the AESO’s profit function can be written as:

$$\begin{array}{l} \Pi =\sum_{t=1}^{T}\left[P_{A,t}D_{t}-\sum_{i}\left(OM_{i}+b_{i}+\tau \varphi_{i}\right)Q_{i,t}+\sum_{k}\left[\left(P_{k,t}-\delta \right)X_{k,t}-\left(P_{k,t}+\delta \right)M_{k,t}\right]\right]\\ \;-\sum_{i}(a_{i}-d_{i})\Delta C_{i},k\in \{BC,MID,SK\}, \end{array}$$ (1)

where Π is profit ($); *i* refers to the generation source (coal, CT gas, wind, etc.); *T* is the number of hours in the one-year time horizon (8760); *D*_*t*_ refers to the load (demand) that has to be met in hour *t* (MW); *Q*_*i*,*t*_ is the amount of electricity produced by generator *i* in hour *t* (MW); *OM*_*i*_ is operating and maintenance cost of generator *i* ($/MWh); and *b*_*i*_ is the variable fuel cost of producing electricity from *i* ($/MWh), which does not change with output (i.e., there are no economies of scale). We define *P*_*j*,*t*_ to be the price ($/MWh) of electricity in each hour, with *j*∈{AB, BC, MID, SK} referring to Alberta, British Columbia, MidC and Saskatchewan, respectively. While Alberta and MidC prices vary hourly, the BC and Saskatchewan prices are fixed at $75 and $56 per MWh, respectively. *M*_*k*,*t*_ refers to the amount imported by Alberta from region *k*∈{BC, MID, SK} at *t*, while *X*_*k*,*t*_ refers to the amount exported from Alberta to region *k*; *δ* is the transmission cost ($/MWh).

The first term in square brackets is simply the gross revenue earned by selling electricity to meet the Alberta load, while the second term refers to the overall costs of internal power generation. Costs are summed across all of the generators; for each generator, it is simply the variable operating & maintenance cost plus fuel cost multiplied by the generator output over the year. (Notice that, because many of the costs required to operate a grid, such as ancillary services, are excluded, it might be more appropriate to refer to Π as gross margin rather than profit.) In addition, the carbon tax paid by each generator is treated as a cost. The carbon tax ($ per tCO_2_) is denoted by τ and is used to incentivize removal of fossil fuel capacity and entry of renewable or nuclear capacity, while *φ*_*i*_ is the CO_2_ emitted when producing a MWh of electricity from generation source *i* (and depends on the fuel source). Then the third term in square brackets refers to the revenue from the sale of exports minus the cost of buying imports, with net exports accounting for the difference between load and internal generation in any hour. The terms in square brackets are then summed over the 8760 hours in the year to which the model is calibrated.

The final term in (1) permits the addition or removal of generating assets, where *a*_*i*_ and *d*_*i*_ refer to the annualized cost of adding or decommissioning assets ($/MW), *C*_*i*_ refers to the capacity of generating source *i* (MW), and Δ*C*_*i*_ is the capacity added or removed. For wind assets, Δ*C*_*W*_ is measured in terms of the number of wind turbines that are added (no reduction in numbers is permitted), each with a capacity of 3.5 MW (as discussed below). Given that wind energy is non-dispatchable (‘must run’), storage is assumed to be available in each period in neighboring jurisdictions via transmission interties; excess energy can be directed or retrieved if the Alberta system cannot respond quickly enough because of extreme variability in wind power output from one period to the next. Further, *R*_*i*_ is the proportion of the capacity of generator type *i* that can to ramped in a given hour; given that the Alberta system can ramp 600 MW of production in any hour, we assume ramp rates of 0.04 for coal and co-gen plants, 0.02 for nuclear plants, and 1.0 for GT gas (peaker) plants. Transmission between Alberta and BC, and Alberta and MidC, is constrained depending on whether power is exported or imported; the import and export constraints are denoted *TRM*_*kt*_ and *TRX*_*kt*_, respectively, with *k* defined above and capacity changing over time for reasons discussed below.

Objective function (1) is maximized subject to the following constraints:

$$\mathrm{Demand is met every hour}:\;\sum_{i}Q_{i,t}+\sum_{k}\left(M_{k,t}-X_{k,t}\right)\geq D_{t},\;\forall t=1,\ldots ,T;k\in \{BC,MID,SK\}$$ (2)

$$\mathrm{Ramping-up constraints}:\;Q_{i,t}-Q_{i,(t-1)}\leq C_{i}\times R_{i},\;\forall i,t=2,\ldots ,T$$ (3)

$$\mathrm{Ramping-down constraints}:\;Q_{i,t}-Q_{i,(t-1)}\geq -C_{i}\times R_{i},\;\forall i,t=2,\ldots ,T$$ (4)

$$\mathrm{Capacity constraints}:\;Q_{i,t}\leq C_{i},\;\forall i,t$$ (5)

$$\mathrm{Import transmission constraint}:\;M_{k,t}\leq TRM_{k,t},\;\forall k,t$$ (6)

$$\mathrm{Export transmission constraint}:\;X_{k,t}\leq TRM_{k,t},\;\forall k,t$$ (7)

$$\mathrm{Non-negativity}:\;Q_{i,t},M_{k,t},X_{k,t}\geq 0,\;\forall t,k,t$$ (8)

In any given hour, electricity can only flow in one direction along a transmission intertie. To model this constraint requires the use of a binary variable for each intertie in the model. To avoid such a nonlinear constraint, we assume that the import and export capacities at any time are equal, and that they equal the total capacity of the line at that time (*TRM*_*k*,*t*_ = *TRX*_*k*,*t*_ = *TCAP*_*k*,*t*_, ∀*k*,*t*), although this applies only to the Alberta-BC intertie where the capacity varies in each period due to internal transmission constraints. These constraints relate, for example, to internal operations that could prevent imported electricity from being delivered to where it is needed. We then employ the following linear constraint to limit the flow of electricity to one direction:

$$X_{k}{}_{,t}+M_{k}{}_{,t}\leq TCAP_{k,t},\forall k,t.$$ (9)

### Wind Data

Hourly wind speed data for 17 locations scattered throughout Alberta were collected from Environment Canada for the decade 2006 through 2015. The location with the highest average wind speed (8.58 m/s) over the period was Pincher Creek in southwestern Alberta, which is about 85 km southwest of Lethbridge, the main center in southern Alberta; Barnwell, which is about 45 km east and somewhat north of Lethbridge, came a distant second with an average wind speed of 4.71 m/s, followed by Raymond (due east of Pincher Creek and about 35 km southeast of Lethbridge), Lethbridge and Killam as the only five sites with average wind speeds above 4.0 m/s. Only Killam is not in southern Alberta as it is located 400 km directly north of Lethbridge.

The power generated by the wind depends not only on wind speed but also on the height of the turbine hub. To determine the actual power available from a wind turbine, the measured wind velocity must be adjusted to obtain wind speed at the turbine hub height. This is done using the following relationship:

$$V_{hub}=V_{data}\times {\left(\frac{H_{hub}}{H_{data}}\right)}^{\alpha },$$ (10)

where *V*_*hub*_ is the wind velocity (m/s) at the turbine hub height, *V*_*data*_ is the measured wind velocity (m/s), *H*_*hub*_ is the height of the wind turbine hub (m), *H*_*data*_ is the height (m) at which the data was measured, and α is the site shear component that is dependent on the type of ground surface on which the wind turbine is built. Empirical evidence suggests that *α* = 0.06 for open water, *α* = 0.10 for short grasses, *α* = 0.14 the most common value, *α* = 0.18 for low vegetation, *α* = 0.22 for forested regions, and *α* = 0.26 for obstructed flows. We use this information to set values of *α* depending on our knowledge of the terrain in the vicinity of the 17 towns in the dataset. (Information on average wind speeds, shear factors employed, and the average power output for the 17 sites is found in Table A–in S1 File; a correlation matrix of wind speeds is found in Table B in S1 File. The power curve for the wind turbine that is used to convert wind speed to power output is provided in Figure A in S1 File. See also www.enercon.de for technical information.) The wind velocity at our sites was measured at 10 m height.

Wind power is related to wind speed as follows:

$$p=½\rho \;v^{3}\pi r^{2},$$ (11)

where *p* is the power of the wind measured in watts, *v* is wind speed measured in m/s, *r* is the radius of the rotor measured in meters, and *ρ* is the density of dry air parameter (assumed equal to 0.94) measured in kg/m^3^. This formula is generally quite useful, but it neglects information on the turbine, particularly the wind speed at which power production begins as well as the cut-out speed where the rotator blade must be turned to avoid damage.

Conversion of the available mechanical energy (wind speed) to electricity is based on the above relations and the technical specifications for a 3.5-MW capacity Enercon E-101 wind turbine. Then, by weighting each location equally, but Pincher Creek at four times the weight of the other locations, we aggregated the potential power production at each location into a single wind power profile for an Alberta-wide, 3.5 MW turbine. The capacity factor of Alberta’s wind regime averaged 28.7% over the 12 years reaching a high of 33.4% in 2013 and a low of 23.3% in 2010; for Pincher Creek, the CF averaged an incredible 55.5%, ranging from 33.9% (2010) to 79.8% (2013). These numbers are potential and not actual CFs. For 2014, our calculations based on wind speed data indicate a CF of 32.7% while the realized CF was 35.6%, although, not surprisingly, most wind farms currently active in Alberta are located in the Pincher Creek region.

**Fig 3:**
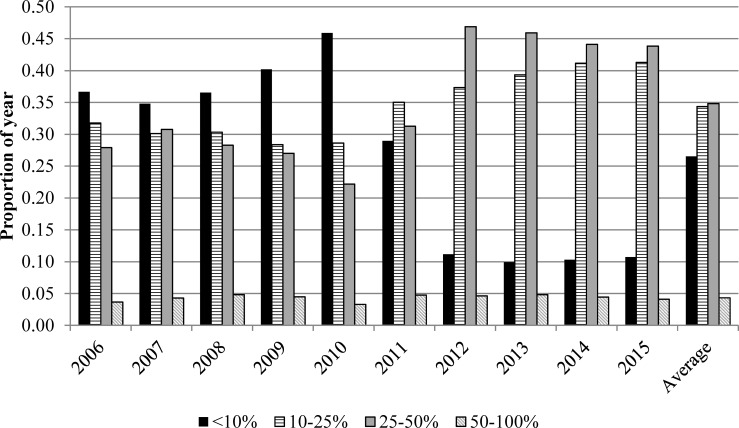
Wind Power Output as a Proportion of Capacity, Alberta, 2006–2015. Source: Authors’ calculations.

As indicated in Fig 3, even if Alberta were to build wind farms across a vast area, about 60% of the time the power produced would be less than one-quarter of the installed capacity. Worse yet, about 96% of the time, wind power would be below half of the rated capacity, and there are only for 17 hours per year on average when the potential electricity available from wind exceeded 75% of capacity. On average, there would be no wind output whatsoever for 5.2 hours during the year, ranging from one hour in 2006 to 13 hours in 2011. No matter how much wind capacity is installed in Alberta, or where it is located, there are times when no wind power is available and many, many times when wind power output is inadequate. In the model, we assume a cap on the number of wind turbines that can be installed of 3500 to avoid potential adverse public reaction related to visual and other dis-amenities and because this would result in installed capacity of 12,250 MW that exceeds peak load.

### Generating Assets

To keep the analysis simple, we ignore marginal generation, such as run-of-river hydro that one subtracts from load in any event, and small amounts of electricity generated from biomass, biogas and flare gas. As evident from Table 2, hydro accounts for 2% and biomass for about 3% of Alberta’s requirements, while other clean energy sources account for negligible power output. Thus, we focus only on coal, natural gas, wind and potentially nuclear energy. Between them, coal and natural gas account for all of the baseload generation, or 63.072 TWh (= 7200 MW × 8760 hours / 1 million), while the remaining 17.19 TWh of electricity is produced by baseload plants, CT gas, wind and imports. Coal plants account for 6258 MW of capacity and co-gen plants for 3892 MW (more than 90% of co-gen plants burn natural gas). For simplicity, we assume that these two sources constitute Alberta’s total baseload capacity (10,150 MW), while remaining capacity consists of 7080 MW of CT gas and 1459 MW of wind (or 417 turbines of 3.5 MW capacity).

**Table 2:** Capacity and Generation, Alberta Electric System, 2014.

	Capacity		Generation	
Fuel Source	MW	Share	GWh	Share
Coal	6,258	38.5%	44,442	55.0%
Natural Gas	7,080	43.6%	28,136	35.0%
Hydro	900	5.5%	1,861	2.0%
Wind	1,459	9.0%	3,471	4.0%
Biomass^a^	447	2.8%	2,060	3.0%
Other^b^	98	0.6%	373	0.0%
**Total**	**16,242**	**100.0%**	**80,343**	**100.0%**

^a^ Co-gen biomass accounts for 158.0 MW of capacity, biogas for 8.8 MW and other biomass for the remainder.

^b^ Includes fuel oil and waste heat, which is a by-product of existing industrial operations with the heat otherwise escaping from an exhaust pipe.

Source: Alberta Utilities Commission (AUC) and Alberta Electric System Operator (AESO)

## Results

The model is parameterized for 2015 –the generation mix, the load and price profiles, and transmission intertie capacities are based on 2015 data from the AESO. However, rather than employing the existing wind profile [11], we employ each of the ten wind profiles that we developed in the previous section. That is, each run of the model provides outcomes for each of the ten wind profiles. We assume that 417 wind turbines are already in place (with a total capacity of 1460 MW), although their wind power profile is different from that of the existing wind farms that have the same capacity. We also note that wind speeds are higher for the period 2012–2015 than for the preceding six years, which turns out to make a large difference as indicated below. We begin by considering the wind speed profile for 2015 only.

In Table 3, we provide the results for the 2015 wind speed profile and several carbon taxes, two levels of the BC-Alberta intertie capacity (storage potential), and whether or not nuclear power is permitted. In the case of the 2015 wind profile, 3226 wind turbines are installed even when there is no carbon tax; that is, the wind profile is such that it pays to install wind power, although, for wind profiles associated with years 2006 through 2011, it is not worthwhile installing any new wind turbines beyond those already in place (417). That is, because the variable costs of wind are effectively zero, whenever there is sufficient wind so that the savings in the variable costs of wind versus other generating assets exceeds the annualized cost of investing in wind then wind will be brought into the generation mix. This occurs for 2012–2015, but not in earlier years.

**Table 3:** Wind versus Nuclear Power in a Carbon Constrained World, Results for the Alberta Electricity Grid, 2015 Wind Profile.

Carbon tax ($/tCO_2_)	Total emissions Mt CO_2_		Trade along intertie (GWh)		Optimal installed capacity (MW)^a^			
		Emission reduction	Import from BC	Export to BC	Coal	Co-gen	Gas	Nuclear
**Base Case Scenario**								
$0	32.74		4,819	54	6,258	3,292	4,184	0
**Current transmission capacity: No nuclear**								
$30	29.05	11.3%	4,829	57	6,258	3,292	6,077	0
$50	29.03	11.3%	4,862	50	0	3,292	6,320	0
$100	28.80	12.0%	4,878	34	0	3,759	6,306	0
**Double transmission capacity: No nuclear**								
$50	25.46	22.2%	9,707	40	0	3,292	7,278	0
$100	25.41	22.4%	9,796	28	0	3,292	6,319	0
**Current transmission capacity: With nuclear**								
$100	8.25	74.8%	4,312	221	0	3,292	4,220	3,728
**Double transmission capacity: With nuclear**								
$100	8.16	75.1%	8,870	232	0	3,292	4,413	3,182

^a^ Under the base case scenario, 2809 additional wind turbines are installed for a total of 3226 turbines (11,291 MW capacity); in all no-nuclear scenarios 3083 additional turbines are installed, for a total of 3500 (12,250 MW). When nuclear power plants are permitted, the existing 417 turbines (1460 MW) remain with no new turbines built.

With a carbon tax and the 2015 wind profile, the optimal number of turbines to install reaches its maximum of 3500. Again, this is not the case for other wind profiles; indeed, only if the carbon tax is $100/tCO_2_ is it worthwhile to increase turbines from 417 to 3500. In particular, for the 2010 wind profile, it is only worthwhile to invest in wind energy if the carbon price is $100; for the $50/tCO_2_ scenario, the number of turbines remains at 417. This has implications as well for the scenario where investment in nuclear power is permitted.

Because our model utilizes all available capacity on the BC-Alberta transmission intertie, wind is encouraged even when there is no carbon tax. Given that imports are the cheapest source of power whenever the internal Alberta price exceeds the fixed BC price, Alberta imports much more along the intertie than it exports. By increasing the variable costs of producing electricity from fossil fuels, the carbon tax exacerbates the import effect because imports are considered to be carbon free. (We employ this assumption because the BC government does not levy its carbon tax on electricity even though power consumed at night likely comes from Alberta coal plants, and even imports from the U.S. could originate from fossil fuel plants.) Hence, as the carbon tax increases in the base scenarios, we see an increase in imports and a reduction in exports (which are taxed when produced by fossil fuel assets).

Now consider the impact of the various wind, carbon tax and nuclear energy scenarios on CO_2_ emissions. Emissions are provided in Table 4 only for the case of the existing capacity constraints on transmission interties. As indicated in the first column, the better wind scenarios (2012–2015) lead to greater investments in wind turbines and lower CO_2_ emissions in order to meet the Alberta 2015 load. With a carbon tax of $30/tCO_2_ there is a significant reduction in emissions, ranging from 6.0% in 2013 (when the wind regime was sufficient to warrant building the maximum 3500 turbines) to 38.0% in 2011 (when no investment in wind energy occurred without incentives). As the carbon tax increases from $30 to $50 and then to $100 per tCO_2_, emission reductions were much smaller reflecting either weak wind regimes or no further potential to add more turbines. Compared to maximum annual emissions of 54.85 Mt CO_2_ (under the weak 2010 wind regime), the best emissions that could be accomplished with a maximum investment in wind energy would occur in 2013, namely, 28.25 Mt CO_2_ –a reduction of 48.5% compared to 2010 baseline emissions. This comparison is invalid, however, since it compares results under different wind regimes. More appropriately, if we look at average annual emissions over the decade, we find that they fell from 45.27 to 31.73 Mt CO_2_, or by only 30%.

**Table 4:** Greenhouse Gas Emissions for Ten Wind Profiles, Various Carbon Taxes, With and Without Nuclear Energy, Mt CO_2_^a^.

	Base	No Nuclear			With Nuclear
Year	$0/tCO_2_	$30	$50	$100	$100
2006	54.58	45.12	38.65	33.95	4.86
2007	54.44	43.70	33.64	33.29	5.77
2008	54.49	45.05	34.00	33.58	5.42
2009	54.59	45.13	40.63	34.16	4.92
2010	54.85	45.32	45.23	35.52	4.80
2011	54.30	33.67	32.82	32.53	6.89
2012	30.59	28.70	28.67	28.51	8.89
2013	30.26	28.46	28.43	28.25	8.83
2014	31.84	28.94	28.91	28.68	8.40
2015	32.74	29.05	29.03	28.80	8.45
**Average**	**45.27**	**37.31**	**34.00**	**31.73**	**6.72**

^a^ Carbon taxes are $/tCO_2_.

The potential for including nuclear energy into the generation mix changes everything. Now average annual emissions fall from 45.27 to 6.72 Mt CO_2_, or by slightly more than 85%. It also turns out that the average costs of reducing carbon emissions is lower under the nuclear option than it is under all of the other options (see Table 5). There are wind regimes and carbon tax scenarios where the cost of reducing emissions is negative, indicating that the tax revenue exceeds the returns to the generators so it is socially beneficial to reduce emissions by investing in wind energy but not privately beneficial. However, costs vary greatly by wind regime and the level of the carbon tax. Therefore, it is necessary to look at the average costs over the decade, which are provided in the last row of Table 5. These indicate that average costs are greater than $800/tCO_2_. In comparison, average costs of reducing CO_2_ emissions under a nuclear option never exceed $500/tCO_2_ and average about $270/tCO_2_.

**Table 5:** Average Costs of Reducing Greenhouse Gas Emissions for Ten Wind Profiles, Various Carbon Taxes, With and Without Nuclear Energy, $/tCO_2_^a^.

	No Nuclear			With Nuclear
Year	$30	$50	$100	$100
2006	$ 742.79	$ 465.74	$ 481.89	$ 190.58
2007	$ 607.88	–$ 64.44	$ 408.25	$ 186.58
2008	$ 89.99	–$ 56.82	$ 436.87	$ 189.13
2009	$ 1,364.39	$ 532.75	$ 754.75	$ 314.53
2010	$ 72.38	$ 906.03	$ 521.85	$ 195.56
2011	$ 167.21	$ 211.08	$ 366.71	$ 167.80
2012	$ 3,606.35	$ 4,125.51	$ 5,233.49	$ 333.36
2013	$ 346.51	$ 901.77	$ 5,512.62	$ 478.38
2014	$ 2,296.96	$ 532.11	$ 3,526.26	$ 428.89
2015	–$ 28.12	$ 1,906.09	$ 2,527.56	$ 390.16
**Average**	**$845.12**	**$864.53**	**$1,806.39**	**$270.45**

^a^ Values are calculated relative to emissions and net returns in the base case. The net present value of the base scenario is subtracted from the NPV for each scenario (minus the associated tax revenue) and then divided by the change in emissions. Negative values indicate that, for the scenario, costs are lower than in the base case.

Surprisingly, compared to other studies [11]; [4], the model results indicate that wind and nuclear energy can coexist, but not in all cases. For the 2011–2015 wind regimes, it would pay to invest in the full complement of 3500 turbines along with an average of about 3600 MW of nuclear power compared to an average of nearly 6500 MW of nuclear capacity for the period 2006–2011 when wind speed regimes led to lower levels of wind power and smaller investments in wind turbines. Indeed, using the 2010 wind regime, it is not worthwhile to invest in new wind capacity while nuclear capacity tops out at 7120 MW.

## Conclusions

To mitigate climate change it is necessary to reduce CO_2_ emissions. Given the importance of electricity to industrial economies, and because of increasing emphasis on using battery-powered, hybrid and/or fuel-cell vehicles that require electricity, there has been a great deal of interest in promoting wind energy. In this study, we examined the potential to replace coal-fired power in Alberta with wind energy. The model used in the analysis is primarily meant to be a policy tool for shedding light on the integration of renewables into electricity grids, and the potential impact on emissions of removing coal-fired power from the generation mix, rather than as a recipe for restructuring the power grid.

Since wind regimes play a very important role, we used wind speed data from locations scattered widely across the province (in some cases a thousand or more kilometers apart) to develop wind power regimes for the decade 2006–2015. Then, using 2015 load and infrastructure data for Alberta, we examined the potential for wind energy to reduce CO_2_ emissions. Our findings indicate that the variability of wind speeds from one hour to the next and from one year to the next is likely to have a great impact on the viability of investments in wind energy. For some wind regimes, investments in wind power make sense without further incentives; indeed, we found this to be the case for the winds that characterize southwestern Alberta, especially around Pincher Creek where most of Alberta’s existing wind farms are located. For other wind regimes, incentives are likely needed to induce investment in wind power. With the exception of certain locations such as Pincher Creek, the variability in wind regimes militates against investment in wind turbines.

We also considered solar power in Alberta but found that, based on the available data, solar power was sufficiently inadequate during winter and night times to warrant consideration at this time. If better data on solar radiation and photovoltaic conversion become available, this will need to be considered further. However, since solar only accounts for some 2% of total renewable capacity and has been shown to have a capacity factor of only 11% in Germany, it is unlikely that solar PV can overcome the problems identified here, particularly the high costs of implementing wind power in areas outside of a small region in southwestern Alberta.

Finally, if politicians in Alberta are serious about reducing greenhouse gas emissions by 30% or more and, at the same time, continue to develop the oil sands despite a cap on annual emissions of 100 Mt CO_2_, it is unlikely this can be achieved without purchasing carbon offsets outside the province or investing in nuclear power. Given that prices of carbon offsets are likely to rise exorbitantly in the future as more and more jurisdictions look to carbon offsets to meet emission reduction targets, and as developing countries are brought into an effective emission-reduction agreement, the most realistic option might well be a nuclear one. Planning should at least consider this option.

## Supporting Information

S1 File Summary of Alberta Wind Data. (DOCX)
