# Supplementary material for: Is There a Future for Nuclear Power? Wind and Emission Reduction Targets in Fossil-Fuel Alberta
Source: PLoS One. 2016 Nov 30;11(11):e0165822. doi: 10.1371/journal.pone.0165822 (PMC5130199; doi:10.1371/journal.pone.0165822)
Supplement: S1 File — (DOCX) [file pone.0165822.s001.docx]

Summary of Alberta Wind Data

Table A: Summary of Wind Speed (m/s), Wind Shear Multiplier and Power Output (MW)

| Location | Average Wind Speed (m/s) | Wind Shear Multiplier (α) | Average Power Output (MW) |
| --- | --- | --- | --- |
| Barnwell  Beaverlodge  Brooks  Fort Vermillion  Grand Prairie  Killam  Lethbridge  Lindbergh  Medicine Hat  Peace River  Pincher Creek  Prentiss  Raymond  Valleyview  Vegreville  Violet Grove  Whitecourt | 4.71  3.02  3.50  1.82  3.35  4.01  4.49  3.13  3.52  2.95  8.58  3.95  4.56  3.90  3.72  2.99  2.66 | 0.10  0.18  0.10  0.22  0.10  0.14  0.10  0.14  0.14  0.20  0.14  0.20  0.10  0.22  0.14  0.10  0.22 | 0.74  0.50  0.39  0.12  0.31  0.61  0.70  0.30  0.49  0.49  1.85  0.73  0.71  0.80  0.28  0.43  0.45 |

The analysis employs an ENERCON E-101 turbine with a nameplate capacity of 3.5 MW. The hub height is 99 m, while the rotor diameter is 101 m (50.5 m radius), with a swept area of 8,012 m^2^. The turbine has three blades and variable rotation speed between 4 and 14.5 rpm, and built-in lightning protection. The power curve is given in Fig S1.

Fig A: Power Curve for ENERCON-101, 3.5 MW Capacity Turbine

Correlation between sites is important to guarantee at least some level of wind output at any time. The wind-speed correlation matrix across sites is provided in Table S2. There are many instances where the correlations of wind speeds across locations exceed 0.60, which confirms that lack of wind is likely to occur at more than one location at the same time. Pincher Creek wind speeds are essentially uncorrelated with the other locations, as are those of Grande Prairie.

Finally, the wind speed data for the Alberta locations by hour for each year over the period 2006-2015, and all the model output results, are available from: [10.6084/m9.figshare.4046904](https://dx.doi.org/10.6084/m9.figshare.4046904)

Table B: Correlation Matrix of Wind Speeds^a^

^a^ Shaded areas indicate correlation exceeds 0.5.
